# Health Disparities Among Asian Immigrants in the United States: The Impact of Arrival Cohorts and Countries of Origin

**DOI:** 10.1093/geroni/igaf122.3951

**Published:** 2025-12-31

**Authors:** Yingzhi Xu

**Affiliations:** Duke University, Durham, North Carolina, United States

## Abstract

Asian Americans are the fastest-growing racial or ethnic group in the United States, having experienced an 81% increase in the population since 2000. Seventy-one percent of adult Asian Americans are immigrants (Pew Research Center, 2022). Much previous research focused on the optimistic social standing of Asian Americans as Asian immigrants’ successful incorporation into American society. However, their health assimilation process and the health disparities among Asian immigrants have been largely underexplored. Using data from the 2008 to 2023 American Community Survey (ACS), this study indicates that the health (cognitive difficulty, physical disability, and independent difficulty, etc.) of Asian immigrants differs by both the arrival cohorts and the sending countries.

